# Spontaneous Retrobulbar Haematoma

**DOI:** 10.1155/2015/796834

**Published:** 2015-05-10

**Authors:** Mehmet Deveer, Nesat Cullu, Halil Beydilli, Hamdi Sozen, Onder Yeniceri, Selcuk Parlak

**Affiliations:** ^1^Department of Radiology, Mugla Sitki Kocman University School of Medicine, 48000 Mugla, Turkey; ^2^Department of Emergency Medicine, Mugla Sitki Kocman University School of Medicine, 48000 Mugla, Turkey; ^3^Department of Infectious Disease, Mugla Sitki Kocman University School of Medicine, 48000 Mugla, Turkey; ^4^Department of Radiology, Private Yucelen Hospital, 48000 Mugla, Turkey; ^5^Department of Radiology, Ankara Numune Research and Training Hospital, 06100 Ankara, Turkey

## Abstract

*Background*. Spontaneous orbital haemorrhage is a very rare condition and vision-threatening event. It may occur due to trauma, orbital surgery/injections, orbital vascular anomalies, and a variety of systemic predisposing factors. Signs of retrobulbar hemorrhage include proptosis, ophthalmoplegia, increased intraocular pressure, loss of pupillary reflexes, and optic disc or retinal pallor. Both Computed Tomography scan and Magnetic Resonance Imaging may be performed in the diagnosis. *Case Report*. A 31-year-old woman was referred to our hospital with a complaint of headache and blurred vision following a strong sneeze. Ophthalmological examination revealed mild Relative Afferent Pupillary Defect in left eye. Computed Tomography revealed left hyperdense retrobulbar mass and displaced optic nerve. T1 weighted hypointense, T2 weighted hyperintense and non-enhanced round shape, sharply demarcated lesion measuring 18 × 15 × 14 × mm in diameter compatible with haematoma was detected by MRI. Surgically Caldwell-Luc procedure was performed. Histological examination confirmed haematoma. Follow-up Magnetic Resonance Imaging revealed a small reduction in the size of lesion but not complete resolution. The patient's complaint was regressed. She is now free of symptoms and is still under surveillance. To our knowledge, this is the first reported case of retrobulbar haematoma caused by sneeze.

## 1. Introduction

Spontaneous orbital haemorrhage is a very rare condition and a vision-threatening event. It may occur due to trauma, orbital surgery/injections, orbital vascular anomalies, and a variety of systemic predisposing factors such as coagulopathy, anticoagulant medications (aspirin, nonsteroidal anti-inflammatory drugs, and Coumadin), dyscrasia (thrombocytopenia, cirrhosis, and leukemia), septicemia, and uncontrolled hypertension [1]. Valsalva maneuver (vomiting and coughing) is another uncommon etiologic factor [2]. Signs of retrobulbar hemorrhage include expanding proptosis, ophthalmoplegia, increased intraocular pressure, loss of pupillary reflexes, and optic disc or retinal pallor [3, 4]. Both Computed Tomography (CT) scan and Magnetic Resonance Imaging (MRI) may be performed in the diagnosis. The treatment aims at lowering intraorbital pressure and protecting the optic nerve from damage. Emergent surgical intervention is the mainstay of treatment. Medical treatment may be considered in cases with minor retrobulbar hemorrhage.

## 2. Case

**Figure 1:**
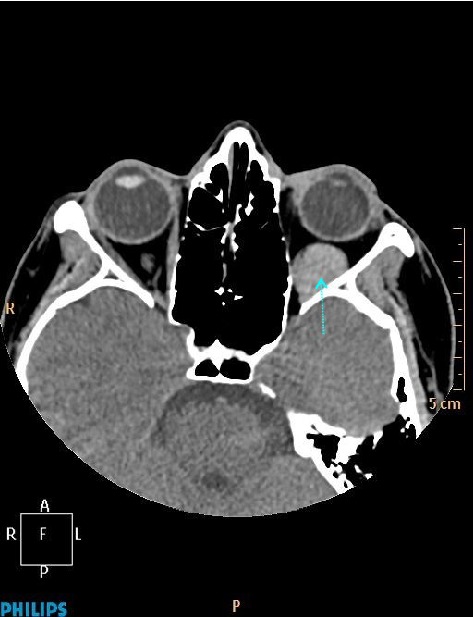
Axial noncontrast CT image showing a hyperdense retrobulbar mass in left orbit (arrow).

**Figure 2:**
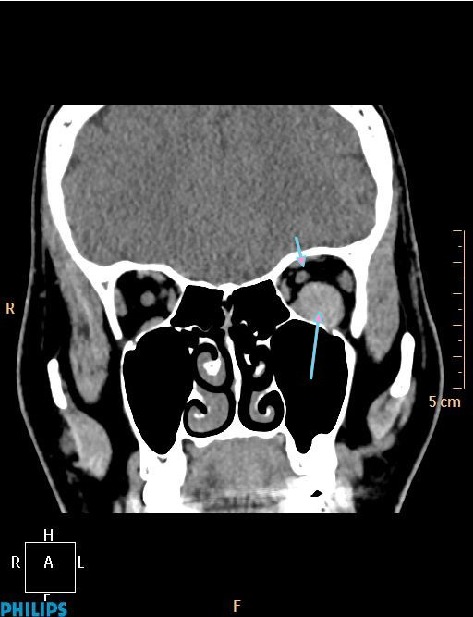
Coronal noncontrast CT image showing hyperdense haematoma (long arrow) and superomedially displaced left optic nerve (short arrow).

**Figure 3:**
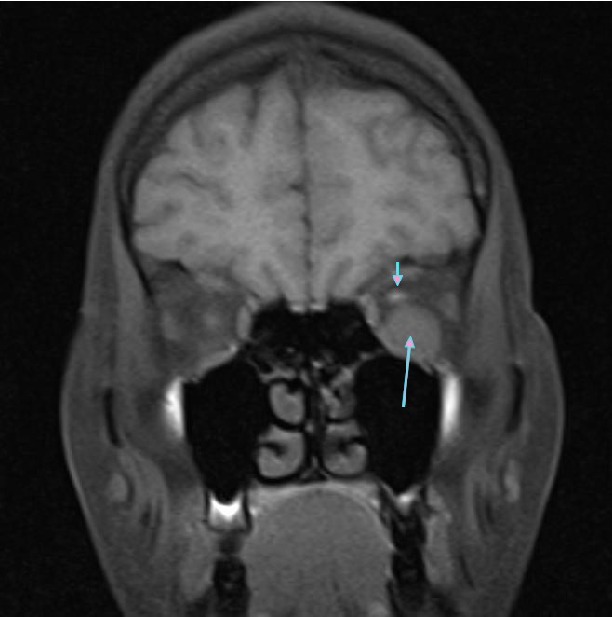
Coronal T1 weighted MR image showing hypointense haematoma (long arrow) and superomedially displaced left optic nerve (short arrow).

**Figure 4 fig4:**
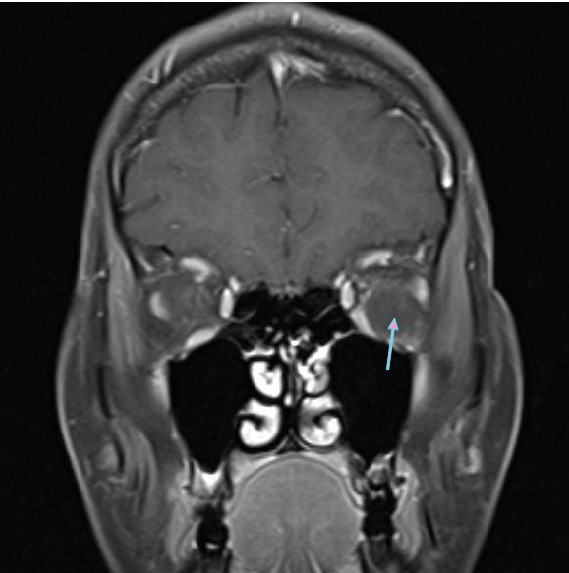
Coronal postcontrast T1 weighted MR image showing nonenhanced hypointense haematoma (arrow).

**Figure 5:**
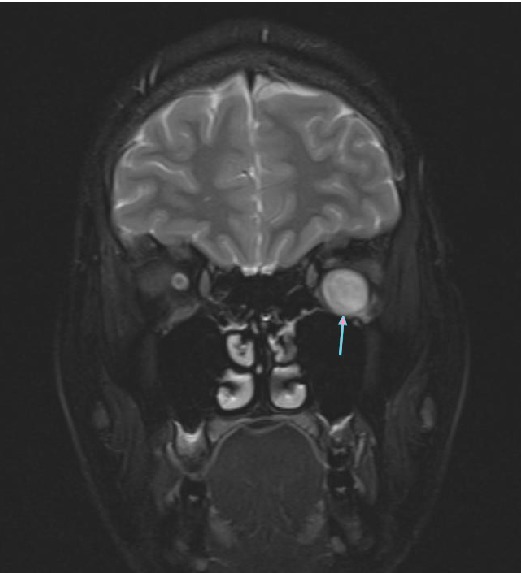
Coronal T2 weighted MR image showing hyperintense haematoma (arrow).

**Figure 6:**
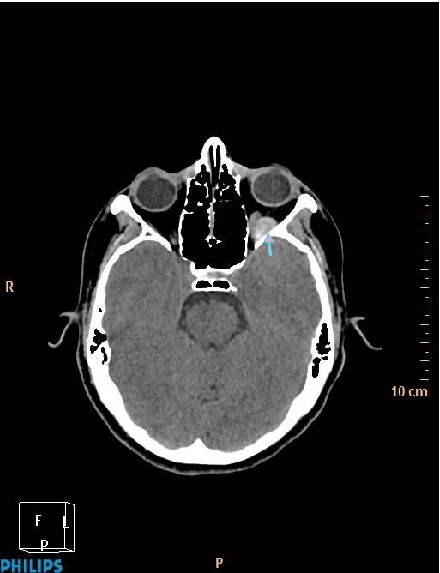
Axial postoperative CT image showing a small reduction in the size of haematoma (arrow).

A 31-year-old woman was referred to our hospital with a complaint of headache and blurred vision following a strong sneeze. She also had nausea and vomiting. Her past medical history was unremarkable. She has no history of alcohol and tobacco consumption. Ophthalmological examination revealed mild Relative Afferent Pupillary Defect (RAPD) in left eye. All blood tests including blood count, electrolytes, coagulation screen, and thyroid function tests were within normal limits. CT revealed left hyperdense retrobulbar mass (70 HU) which displaced optic nerve superomedially (Figures 1 and 2). For better visualization, T1 weighted hypointense, T2 weighted hyperintense and non-enhanced round shape, sharply demarcated lesion measuring 18 × 15 × 14 × mm in diameter competible with haematoma was detected by MRI (Figures 3 [Fig fig4]–5). Surgically Caldwell-Luc procedure was performed. Histological examination revealed blood clot, adipose tissue, connective tissue with no evidence of malignancy, and inflammatory cell infiltration consistent with haematoma. One day after the procedure, follow-up MRI (Figure 6) revealed a small reduction in the size of lesion (15 × 7 × 9 mm) but no complete resolution was detected. The patient's complaint was regressed. She is now free of symptoms and is still under surveillance.

## 3. Discussion

Spontaneous retrobulbar haematoma is rare. The largest report of nontraumatic orbital hemorrhage describes 115 cases over a 24-year period and revealed that orbital vascular malformations (orbital varix, lymphangioma, or arteriovenous malformation) were present in 90%, had additional or other predisposing factors in 11% (childbirth, prolonged headstands, hypertension, or coagulopathies), and had no predisposing factor in 5% [1]. We did not detect any predisposing factor including vascular malformations and hypertension or coagulopathies.

The most common symptoms of retrobulbar hemorrhage include pain, pressure, and loss of vision. Other common symptoms include diplopia, nausea, and vomiting [2, 5]. The pain associated with retrobulbar hemorrhage is typically severe [3]. Our patient also had severe headache, blurred vision, nausea, and vomiting.

RAPD is an extremely significant clinical finding. Optic nerve lesions and severe retinal disease are most common factors RAPD [6]. We detected RAPD and we thought this is the cause of optic nerve compression.

Valsalva maneuver has been reported to be the cause of nontraumatic orbital hemorrhage in patient with no known underlying vascular malformation [7]. In these cases pathophysiologic mechanism is thought to begin with an increase in intra-abdominal and intrathoracic pressure against a closed glottis. Since orbital veins do not contain valves, elevated intraabdominal pressure results in congestion and ruptur. In our case, the Valsalva maneuver has been reported to cause subconjunctival hemorrhage, hemorrhagic retinopathy, and periorbital petechiae [8]. Strong sneeze made a effect similar to valsalva maneuver.

CT or MRI is necessary for information about normal orbital structures and pathological lesions. Both of them confirm the diagnosis and exclude other potential causes. Due to the emergent nature of retrobulbar hemorrhage in cases associated with trauma, CT is preferred because of its fast and better visualization of the bony anatomy. The other conditions including vascular anomalies, MRI provide better information [9]. We initially performed CT, and, after detecting mass, MRI was performed for diagnosis.

The surgical approach was limited to simple drainage of the collection. If the patient has diffuse bleeding causing compressive optic neuropathy or encysted collection of blood with compressive optic neuropathy, urgent surgical intervention was performed. We performed surgical treatment cause of compressive optic neuropathy.

In the literature, we find that sneeze leads to orbital haematoma. To our knowledge, this is the first reported case of retrobulbar haematoma caused by sneeze. That is why we wanted to share it with our colleagues.
